# Community Care Administration of Spinal Deformities in the Brazilian Public Health System

**DOI:** 10.6061/clinics/2017(08)06

**Published:** 2017-08

**Authors:** Mario Bressan-Neto, Carlos Fernando Pereira da Silva Herrero, Lilian Maria Pacola, Altacílio Aparecido Nunes, Helton Luiz Aparecido Defino

**Affiliations:** I Departamento de Biomecanica, Medicina e Reabilitacao do Aparelho Locomotor, Faculdade de Medicina de Ribeirao Preto, Universidade de Sao Paulo, Ribeirao Preto, SP, BR; II Departamento de Medicina Social, Faculdade de Medicina de Ribeirao Preto, Universidade de Sao Paulo, Ribeirao Preto, SP, BR

**Keywords:** Spine, Waiting Lists, Scoliosis, Spinal Curvatures, Healthcare Financing

## Abstract

**OBJECTIVE:**

Underfunding of the surgical treatment of complex spinal deformities has been an important reason for the steadily growing waiting lists in publicly funded healthcare systems. The aim of this study is to characterize the management of the treatment of spinal deformities in the public healthcare system.

**METHODS:**

A cross-sectional study of 60 patients with complex pediatric spinal deformities waiting for treatment in December 2013 was performed. The evaluated parameters were place of origin, waiting time until first assessment at a specialized spine care center, waiting time for the surgical treatment, and need for implants not reimbursed by the healthcare system.

**RESULTS:**

Ninety-one percent of the patients lived in São Paulo State (33% from Ribeirão Preto - DRS XIII). Patients waited for 0.5 to 48.0 months for referral, and the waiting times for surgery ranged from 2 to 117 months. Forty-five percent of the patients required implants for the surgical procedure that were not available.

**CONCLUSION:**

The current management of patients with spinal deformities in the public healthcare system does not provide adequate treatment for these patients in our region. They experience long waiting periods for referral and prolonged waiting times to receive surgical treatment; additionally, many of the necessary procedures are not reimbursed by the public healthcare system.

## INTRODUCTION

In the Brazilian Unified Health System (SUS), patients with complex spinal deformities are referred to specialized tertiary care spine centers to receive complex treatments according to regulating norms 1. Patients who need surgical correction of the deformity are then placed on the surgery waiting list. Although the number of patients is relatively small compared to those of other specialties, the surgical treatment of spinal deformities has characteristics that interfere with the dynamics and flow of treatment. The surgeries have a long duration and require experienced surgeons with specialized deformity surgery training, high-cost implants and technical resources, and there are frequent readmissions and reoperations due to the high percentage of complications associated with the treatment 2. The costs of the treatment are not fully reimbursed by the public health system 3. Therefore, each procedure may lead to a financial deficit for the institution performing the surgical treatment. Due to the lack of financial resources, necessary surgeries have not been performed according to actual clinical needs.

Due to underfunding, the surgical treatment of complex spinal deformities in publicly funded healthcare systems has been subjected to steadily lengthening waiting lists 4-7. The long waiting times for deformity correction have been associated with clinical and radiological worsening of the deformity, reduced corrective potential, the need for more complex procedures, increased morbidity and complication rates, increased anxiety of patients and parents and lower scores on health-related quality of life questionnaires 8-11. Therefore, the increase in waiting times for treatment has resulted in higher treatment costs 12,13, revealing the paradoxical manner in which the health system has managed the treatment of patients with complex spinal deformities.

The present study was designed after considering the current difficulties related to the underfunding of the surgical treatment of spinal deformities within the publicly funded healthcare system, including the progressive increase in the number of patients on the waiting list for surgery and the gradual decrease in the number of surgeries being performed. The objective of the study was to analyze and characterize a sample of patients with complex spinal deformities waiting for surgical treatment at a referral center.

## MATERIALS AND METHODS

The study was approved by our Institutional Review Board (process number 833,475). It was designed as a cross-sectional study to evaluate the clinical, epidemiological and demographic characteristics of patients on a waitlist for the surgical treatment of spinal deformities. Sixty patients with pediatric spinal deformities (40 females – 66.7%) who were waiting for surgical treatment of these deformities on December 31, 2013, at the Orthopedic Department of a tertiary specialized spine care center were included. Patients with adult or degenerative deformities and patients with inconsistent medical record data were excluded.

The ages of the patients ranged from 3 to 23 years old (mean 13.5±3.7 years old). The etiologies of the deformities included the following: neuromuscular, congenital, idiopathic, and syndromic conditions, Marfan syndrome, and neurofibromatosis.

The following parameters were evaluated: age, gender, etiology, origin, method of referral, waiting time until first assessment at a specialized spine care center, treatment received before referral, waiting time for surgical treatment, requirement for implants not reimbursed by the healthcare system (iliac screws, pediatric implants, cervicothoracic-transition implants, Vertical Expandable Prosthetic Titanium Rib - VEPTR®), and whether treatment was sought at other specialized spinal deformity care centers.

All collected data were stored in *Microsoft Excel^®^* (Microsoft Corporation, Redmond, WA, EUA), checked for consistency and analyzed and compared using SAS JMP^®^ (SAS Institute Inc., Cary, NC, EUA). Data with a normal distribution were described by means and standard deviations and compared using Student’s T-test; data with non-parametric distributions were described by the median and interquartile ranges (IQR) and compared using analysis of variance (ANOVA) and the Mann-Whitney U-test (intergroup analysis). Paired Student’s T-tests were used for the intragroup analysis. The significance level (α) was set at 0.05.

## RESULTS

**Figure 1:**
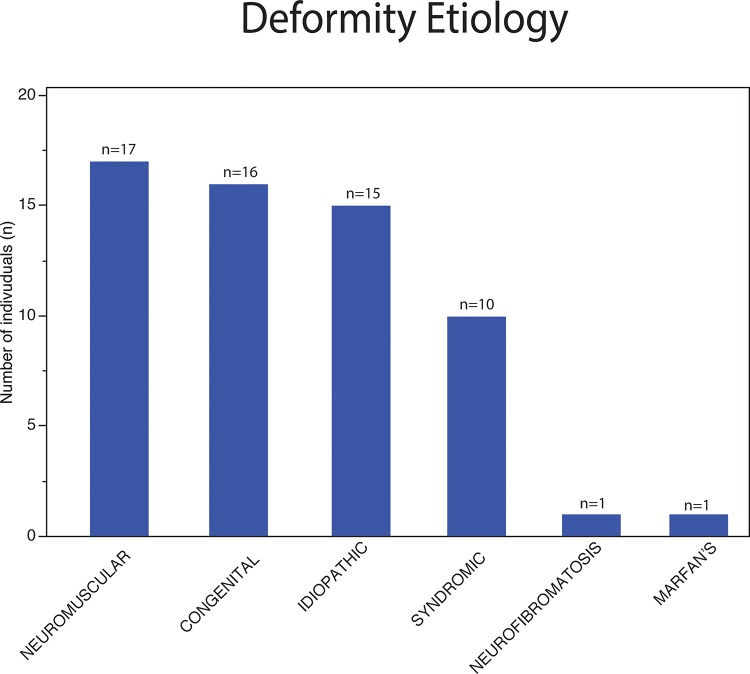
Etiology of the deformities of patients on the waiting list for surgical treatment.

**Figure 2:**
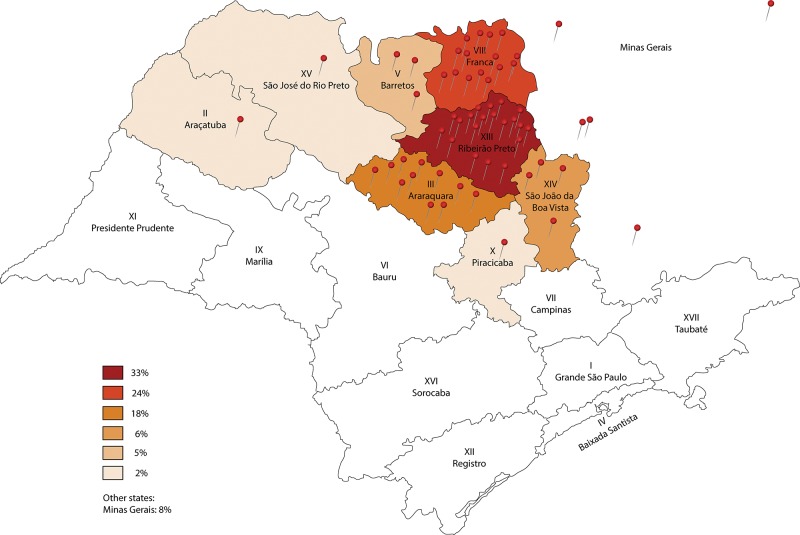
Origin of the patients waiting for spinal deformity surgical treatment in December 2013. The map represents the health care regional divisions of Sao Paulo State, and each pin represents one patient.

On December 31, 2013, 60 patients (40 female – 66.7%) were on the waiting list to receive surgical treatment for spinal deformities at our institution; these patients were a mean age of 13.5±3.7 years old (range, 3–23 years old). The deformity etiologies included the following: neuromuscular (17 patients – 28.3%), congenital (16 patients – 26.7%), idiopathic (15 patients – 25.0%), and syndromic (10 patients – 16.7%) conditions, Marfan syndrome (1 patient – 1.7%), and neurofibromatosis (1 patient – 1.7%) (Figure 1). Six of these patients had isolated sagittal plane deformities (10%). The patients originated mainly from the region of Ribeirao Preto and the surrounding regions (91.7%) (Figure 2). Only 15 patients (25%) received primary care treatment (for example, bracing).

**Figure 3:**
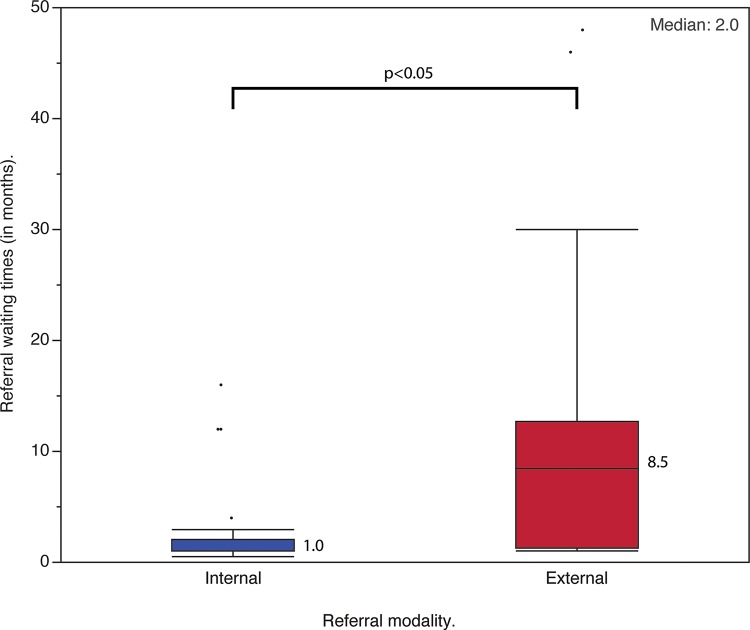
Boxplot representing the waiting times for internal and external referrals for specialized spine care consultation. The boxes represent the interquartile range (IQR); the whiskers represent the first quartile minus 1.5 times the IQR and the third quartile plus 1.5 times the IQR. The dots represent outliers. The numbers next to the boxplots represent the median of internal and external referrals, and the number in the top-right corner represents the median of the total referral waiting times.

The patients on the waiting list were referred to our specialized spine care center by the State Department of Health (31 patients – 51.7%) and by internal referrals from other specialties within our hospital (29 patients – 48.3%). The time between referral and first assessment ranged from 0.5 to 48.0 months (median, 2 months; IQR, 11.0 months). External referrals required a median of 8.5 months (IQR, 11.5 months) of waiting until the first assessment, whereas internal referrals required a median of one month (IQR 1 month) (*p*=0.0013 – Figure 3).

At the first visit, 30 patients (50%) had a formal surgical indication. Twenty-seven patients (45%) required unavailable implants for the surgical procedure. More neuromuscular patients needed non-available implants (70.6%), followed by congenital (62.5%), syndromic (40.0%) and idiopathic (3.7%) patients (Table 1).

**Figure 4:**
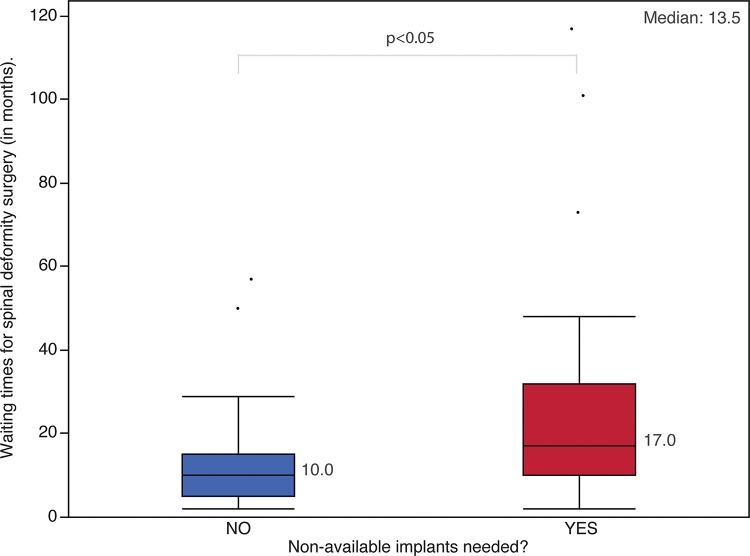
Waiting times for the surgical treatment of spinal deformities and the requirement of non-available implants. The boxes represent the interquartile range (IQR); the whiskers represent the first quartile minus 1.5 times the IQR and the third quartile plus 1.5 times the IQR. The dots represent outliers. The numbers next to the boxplots represent the median of each group, and the number in the top-right corner represents the median of the total waiting times for surgery.

As of December 31, 2013, the 60 patients with pediatric spinal deformities had waited a median of 13.5 months on the waitlist (IQR, 13.8 months; range, 2–117 months). A significant difference was observed in the waiting times between the patients who required unavailable implants (median, 17 months; IQR, 22 months) and those who required available implants (median, ten months; IQR, ten months) (*p*=0.009) (Figure 4). While waiting for surgery, seven patients (11.7%) visited other spine deformity treatment centers to try and receive adequate treatment; only one was successful and was treated after 13 months of waiting. The reason that the other institutions denied treatment was the excessive number of patients on the waiting list.

## DISCUSSION

The results from this group of patients with spinal deformities waiting for surgical treatment illustrate some well-known problems faced by health care professionals who work in this field and may serve as guidance for providers facing similar issues. The SUS was created in 1990, after the 1988 Brazilian constitution recognized health as a citizen’s right and a duty of the state 14. It was developed based on the doctrinal principles of universality, equity, and integrality. As a strategy to achieve equity, the organizational principles of regionalization and hierarchization were implemented. According to these principles, patients should be evaluated at primary care centers located as close as possible to their homes 15. Primary health care centers should provide universal access to care as well as comprehensive health care, health promotion and disease prevention efforts. When further specialized care is needed, patients should be referred to more complex levels of care. Secondary care centers are responsible for medium complexity procedures, predominantly specialist outpatient care and procedures, and tertiary health care centers are responsible for the most complex treatments, including high-cost treatments 14. The patients in our study were treated according to the SUS regulation norms 1,15; however, this approach has not been effective.

Most patients originated from regional divisions near the specialized spinal care centers, characterizing the homogeneity of the population referred for surgical treatment. This distribution indicates that the patients are being referred based on the health care system’s regulation norms and the regionalization principle 15. Five patients (8.3%) from distant regions originally sought treatment in their local region without success, but those cases were not sufficient to characterize noncompliance with this regionalization principle.

The prevalence of deformity etiologies in the studied group diverged from that of the general population 16. Idiopathic scoliosis is the most prevalent pediatric deformity, affecting 2.2% to 4.3% of schoolchildren in Brazil 17,18. According to the Scoliosis Research Society (SRS), idiopathic scoliosis represented 58% of the 19,360 patients who underwent surgical correction between 2004 and 2007, followed by neuromuscular (24%) and congenital deformities (10%) 16. The predominance of neuromuscular and congenital deformities in the studied group of patients characterizes the complexity of patients referred for treatment who continue to wait for resolution.

The observed waiting times until first assessment at a specialized spine care center demonstrate the lack of efficiency in the referral of patients with spinal deformities in our region. External referral waiting times were 8.5 times longer than internal referral waiting times. Wright et al. 6, based on expert consensus, determined that the ideal waiting time for referrals of patients with spinal deformities was less than six months. Additionally, only 25% of the patients had received treatment at a primary care center, and 50% of the patients referred for evaluation had a formal surgical indication at the first assessment. These observations suggest that the hierarchical principles of the SUS are not efficient for the treatment of patients with spinal deformities in our region. These patients do not receive appropriate treatment at primary care centers and are subjected to unacceptably long waiting times to be evaluated at tertiary care centers. When surgical treatment is indicated at a tertiary care center, almost half of the patients (45%) require implants that are not reimbursed by the public health care system (SUS) and are therefore waiting for a modality of treatment that is not available.

The median waiting time for patients on the waiting list for surgery was 13.5 months, which is beyond the limits established in other countries with publicly funded health care systems. The mean waiting time for surgery is five to nine months in the United Kingdom 5, six to 12 months in Canada 5,8 and 11 months in New Zealand 4. These data show that long waiting times exist for the surgical treatment of spinal deformities in many publicly funded health care systems. However, our study demonstrated that this issue is more severe in the Brazilian SUS. It is important to emphasize that unlike other studies 4,5,8, which measured waiting times until surgery was performed, our waiting times were obtained from a cross-sectional study. Our waiting time data reflect the median time that individuals had waited for surgery as of December 31, 2013, not how long they will wait for surgery; therefore, our data underestimate the waiting times for surgery.

The long waiting times for the surgical treatment of spinal deformities prompted a reformulation of the health care policies in Canada. Wright et al. 6 determined that as soon as the decision for surgery is made, the maximum acceptable waiting time is six months. Ahn et al. 8 suggested that the ideal maximal waiting time would be even less (three months) because patients waiting longer for surgery had higher complication rates and because a greater number of procedures than initially planned was often required. To achieve this goal, they concluded that resource allocation would be necessary to increase the availability of operating rooms, capable surgeons, hospital beds and reimbursement. As a result, the overload of the health care system would decrease by minimizing the number of complications and additional procedures.

The results of this study revealed important aspects of the surgical treatment of spinal deformities and indicated that the present model of management does not result in adequate treatment of these patients in our region. They experience long waiting periods to receive referrals, specialized spine care assessment, and definitive surgical treatment. Nevertheless, our study demonstrated that almost half of the patients were waiting for a treatment modality that was not available. We hypothesize that these issues may be present in most specialized spine care centers; however, further studies are needed to examine the extent of the problem. We concluded that the management of patients with pediatric spinal deformities must be reviewed and modified to ensure that patients receive adequate treatment without an excessive delay of surgical treatment.

## AUTHOR CONTRIBUTIONS

Bressan-Neto M collected and analyzed the data and wrote the manuscript. Herrero CF helped with the writing of the manuscript and with the statistical analysis. Pacola LM helped with data acquisition and manuscript revisions. Nunes AA helped designing the study, focusing mainly on the socio-demographic and epidemiological aspects, supervised the statistical analysis and helped writing the manuscript. Defino HL conceived the study, participated in its design and coordination, and helped with writing and revising the manuscript. All authors read and approved the final version of the manuscript.

## Figures and Tables

**Table 1:** Distribution of patients requiring non-available implants and the etiology of their spinal deformities.

Etiology	Absolute Number	Percentage
Neuromuscular	12	70.6%
Congenital	10	62.5%
Syndromic	4	40.0%
Idiopathic	1	3.7%
**Total**	**27**	**45.0%**

* Percentage of patients relative to the total in specific etiologic groups.
